# An AI-based approach driven by genotypes and phenotypes to uplift the diagnostic yield of genetic diseases

**DOI:** 10.1007/s00439-023-02638-x

**Published:** 2024-03-23

**Authors:** S. Zucca, G. Nicora, F. De Paoli, M. G. Carta, R. Bellazzi, P. Magni, E. Rizzo, I. Limongelli

**Affiliations:** 1 enGenome Srl, 27100 Pavia, Italy; 2 Department of Electrical, Computer and Biomedical Engineering, University of Pavia, Pavia, Italy; 3 University of Pavia, 27100 Pavia, Italy

## Abstract

Identifying disease-causing variants in Rare Disease patients’ genome is a challenging problem. To accomplish this task, we describe a machine learning framework, that we called “Suggested Diagnosis”, whose aim is to prioritize genetic variants in an exome/genome based on the probability of being disease-causing. To do so, our method leverages standard guidelines for germline variant interpretation as defined by the American College of Human Genomics (ACMG) and the Association for Molecular Pathology (AMP), inheritance information, phenotypic similarity, and variant quality. Starting from (1) the VCF file containing proband’s variants, (2) the list of proband’s phenotypes encoded in Human Phenotype Ontology terms, and optionally (3) the information about family members (if available), the “Suggested Diagnosis” ranks all the variants according to their machine learning prediction. This method significantly reduces the number of variants that need to be evaluated by geneticists by pinpointing causative variants in the very first positions of the prioritized list. Most importantly, our approach proved to be among the top performers within the CAGI6 Rare Genome Project Challenge, where it was able to rank the true causative variant among the first positions and, uniquely among all the challenge participants, increased the diagnostic yield of 12.5% by solving 2 undiagnosed cases.

## Introduction

Genomic variant interpretation is a complex process whose aim is to identify pathogenic events. Genetic diagnosis is pivotal for the treatment of Rare Diseases, a heterogeneous set of disorders that affect approximately 6% of the Western population and in which 80% of cases are estimated to be caused by inherited variants (Genomes Pilot on Rare-Disease Diagnosis in Health Care — Preliminary Report 2021). Even with the technological advances introduced by Next Generation Sequencing (NGS) technologies, as of today Rare Diseases diagnostic yield greatly varies from 35 to 55%, depending on the disorder (Genomes Pilot on Rare-Disease Diagnosis in Health Care — Preliminary Report 2021), leaving about 200,000 million patients without a clear diagnosis (Vinkšel et al. 2021).

In 2015, the American College of Medical Genetics and Genomics (ACMG) together with the Association for Molecular Pathology (AMP) have defined a set of criteria and rules to interpret germline variants in a five-tier system (Richards et al. 2015). These standard guidelines consider different information related to germline variants, such as family segregation, in silico prediction of damaging impact, and past interpretation from reliable sources, to eventually classify a genomic variant into one of the following classes: Pathogenic, Likely pathogenic, Benign, Likely benign and Variant of Uncertain Significance (VUS). Over the years, the ACMG/AMP guidelines have become the standard for germline variants interpretation worldwide, and several computational approaches, both commercial or not, have been proposed to automatically implement this complex set of rules (Li and Wang 2017; Scott et al. 2019; Nicora et al. 2018; Whiffin et al. 2018; Xavier et al. 2019; Ravichandran et al. 2019; Peng et al. 2021; Kopanos et al. 2019), thus supporting clinicians in variant interpretation. Despite the great importance of the ACMG/AMP guidelines, class-based systems may not be effective in clinical settings, when thousands of variants per proband need to be examined to identify few causatives. Additionally, these guidelines are intended to determine variant pathogenicity, an assessment that should be independent of interpreting the cause of the disease for a given patient (Richards et al. 2015). In this context, variants should be evaluated not only given their potential pathogenicity but also in light of a patient’s phenotypes and family information. The assessment of variant pathogenicity is indeed distinct from interpreting the cause of a disease in individual patients. According to the study by Shen et al., the response of patients to specific therapies targeted at presumed genetic diseases can serve as a valuable criterion for clarifying the pathogenicity of genomic variants. This approach underscores the importance of evaluating variant pathogenicity independently, without conflating it with the overall diagnosis of the disease’s cause in patients (Shen et al. 2021). Variant filtering and variant prioritization can extremely reduce clinicians’ burden, by reducing the number of variants that need to be evaluated and pinpointing the most interesting variants in the top positions after prioritization. Few attempts have been made to translate the ACMG/AMP five-tier system into a score-based system (Nicora et al. 2018; Tavtigian et al. 2018; Tavtigian et al. 2020), but, to the best of our knowledge, they have not been extensively evaluated in the context of variant prioritization (VP). On the contrary, many computational tools performing a phenotype-based variant (or gene) prioritization have been developed. These approaches rank variants based on the likelihood that they can impact their gene’s function and that the mutated gene can cause the phenotype(s) observed in the patient. To represent patient’s phenotypes in a standardized way, most VP tools encode phenotypes in terms of Human Phenotype Ontology (HPO) terms (Köhler et al. 2021). The HPO is a standardized vocabulary containing thousands of phenotypic abnormalities associated with diseases. HPO expansion and optimization have been a key driver for the development of VP tools (Yuan, et al. 2022), most of which are machine learning based. Most of these tools incorporate both HPO terms and in silico prediction of variant damaging impact, such as CADD (Rentzsch et al. 2021) cadd of REVEL (Ioannidis et al. 2016), while ACMG/AMP criteria are exploited as features for the machine learning model by XRare (Li et al. 2019). Several studies have performed a comparison of VP tools in terms of prioritization ability. Solved cases from large studies, such as the 1000 Genomes Project (Auton et al. 2015) or the Deciphering Developmental Disorders (DDD) project (Firth 2011), have been exploited in different benchmark analysis (Yuan et al. 2022; Jacobsen et al. 2022). Others have used data from smaller set of real patients (Kelly et al. 2022; Pengelly et al. 2017; Tosco-Herrera et al. 2022), or from simulated patients (Smedley et al. 2015) to assess VP tools prioritization ability.

In this context, the Critical Assessment of Genome Interpretation (CAGI) plays an important role in assessing performance and defining the state-of-the-art of computational tools for genomic variant interpretation. Within the CAGI challenges, participants (both academic and industry) are provided with genomics data to develop their approaches for variant interpretation, which are subsequently evaluated by the CAGI consortium (TCA 2022). In 2021, the CAGI consortium proposed a challenge to identify diagnostic variants in Rare Diseases from the Rare Genome Project (RGP). Challenge participants were given a set of families and proband-only cases, along with the Human Phenotype Ontology (HPO) terms associated with the affected probands. The aim of the challenge is to rank the predicted disease-causing variants according to the probability of causation so that a molecular diagnosis can be established, and optionally provide a measure of uncertainty of the prediction. The dataset consists of both solved and unsolved cases, as determined by a team from the Broad Institute, and, when possible, confirmed by clinicians. The solved cases were used to evaluate participants’ performance, while the unsolved cases were included to identify new potential causal variants. Participants were invited to submit up to six different predictions for each case.

Recently, the results of the CAGI6-RGP challenge have been published as a pre-print by Stenton et al. (Stenton et al. 2023). According to this manuscript, 5 different groups have been disclosed as participants in this challenge. They all applied a different solution for phenotype-driven variant prioritization.

The commercial solution developed by Invitae Moon is based on the automated prioritization of variants based on clinical and genetic data. This system is based on an internally curated and up-to-date gene-phenotype association database (Apollo). Katsonis et al. proposed a solution based on the Evolutionary Action method that predicted the functional consequences of missense variants (Katsonis and Lichtarge 2014). This solution was combined with a phenotype-driven approach and accounts also for variant’s quality and frequency in population databases, even if the full method has not been published yet. The TCS group used in-house tools for variant prioritization (VPR) and gene prioritization (PRIORI-T (Rao et al. 2020) and GPrio). Another popular tool used within this context for phenotype-driven variant prioritization is the open-source Exomiser (Bone et al. 2016).

We have participated in the CAGI6 RGP challenge submitting four different predictions, both integrating ACMG/AMP guidelines for variant interpretation, phenotypic similarity, family segregation, and expected inheritance for the condition. Our best model was recognized as a best-performing approach in variant prioritization for the diagnosed samples in the test set, among 52 submitted models. Moreover, it was able to uniquely identify a deep intronic ASNS variant in an unsolved case. This variant was subsequently evaluated to be the causative variant for that patient. Additionally, a new causative near splice variant was identified in TCF4. Both diagnoses were returned to the patients, thus increasing the diagnostic yield by 12.5%.

## Materials and methods

### Dataset preprocessing

CAGI6 RGP organizers shared with all participants 35 solved cases for training (6 cases proband-only, the remaining trios, duos, and quartets). For each subject, the VCF file was provided. HPO terms and the known causative variant for each proband were also shared. A detailed description of proband’s phenotypes, causative variants and family composition is reported in Challenge datasets in Stenton et al. (Stenton et al. 2023).

As a preprocessing step, we filtered out variants with high allele frequency in gnomAD (common variants in populations with allele frequency greater than 0.05 according to gnomAD v3.0) and sequencing artifacts, i.e. variants detected in more than 13 alleles in the whole dataset provided within the challenge.

### Ethical considerations

All RGP participants engage in a consent video or video call with a trained research coordinator. During this interaction, participants review the study protocol, which includes provisions for sharing de-identified data, and they provide signed informed consent (Mass General Brigham IRB protocol 2016P001422). The RGP organizers executed an institutionally signed (Broad-Northeastern) data transfer agreement. As predictors in the CAGI 6 challenge, we were required to sign and adhere to a registered access model and the CAGI Data Use Agreement (genomeinterpretation.org/data-useagreement.html). All details are provided in Stenton et al. (Stenton et al. 2023).

### Features engineering

We developed our model to emulate the reasoning process of a geneticist overseeing the Variant Interpretation process, aimed at solving undiagnosed cases. During the machine learning model’s feature engineering phase, each feature was meticulously designed to ensure independence from both the disease and the associated phenotypic characteristics of the patient. To accurately mimic the real diagnostic workflow, we identified four essential levels of information necessary to determine whether a specific variant constitutes the molecular diagnosis for the patient.

The first level involves assessing the pathogenicity of the variant (as an example, according to the ACMG/AMP guidelines). This question can be addressed by determining the variant’s pathogenicity through a quantitative pathogenicity score.

The second level revolves around determining whether the variant explains the observed phenotypes in the patient. Quantitative measures of phenotypic similarity between the variant and the proband’s phenotypes are employed to answer this question.

The third level focuses on evaluating whether the variant segregates in the family based on a complete penetrance model. Additionally, it examines whether the observed segregation aligns with the inheritance pattern associated with the condition. In this case, it is essential to verify the actual or inferred segregation pattern and the expected inheritance patterns for the disease.

Finally, the variant must be reliable and not be a result of sequencing artifacts or a common variant in the studied population. Variant quality metrics can be leveraged to address this aspect.

This set of information will serve as features for our model.

By considering these four levels of information, our model emulates the reasoning process of a geneticist, aiding in the interpretation of variants and potentially leading to the identification of the molecular diagnosis for undiagnosed cases.

The list of features used in the model is reported in Table 1.

**Table 1:** Summary of the features exploited by the Suggested Diagnosis model

Feature	Description	Type
Variant pathogenicity	ML-based eVai pathogenicity score, described by Nicora et al. (Nicora et al. 2022a)	Probability of pathogenicity between 0 and 1
Phenotypic similarity	Similarity between a patient’s clinical manifestations and disease descriptions associated with genes (based on the Human Phenotype Ontology)	Continuous
Inheritance information	Match between the variant segregation pattern in the family and the expected inheritance for the associated condition	Boolean
Variant quality	Whether the variant has good quality or not	Boolean

### Variant pathogenicity

To assess variant pathogenicity, we analyzed the VCFs with eVai, the enGenome proprietary software that implements the ACMG/AMP guidelines. eVai assigns one of the five ACMG/AMP classes to each variant according to the implementation previously shared (Nicora et al. 2018). Additionally, eVai associates to each variant a pathogenicity score, which is predicted by a ML classifier based on the ACMG/AMP criteria triggered by each variant. We have previously developed a logistic regression model to predict variant pathogenicity based on ACMG/AMP standard guidelines (Nicora et al. 2022a), where the model was trained on bona-fide pathogenic and benign variants from the Clinvitae database. Hence, we assign the logistic regression predicted probability of pathogenicity (named *pathogenicity score*) to each variant. The ACMG/AMP classification is computed for all the variants on disease-associated genes, according to the principal databases, such as MedGen (https://www.ncbi.nlm.nih.gov/medgen/), Disease Ontology (https://disease-ontology.org/), and Orphanet (https://www.orpha.net/).

In our previous work, we showed that the eVai pathogenicity score had better performance in comparison with CADD, VVP, and a Bayesian approach on data from Clinvitae and the ICR639 project (Nicora et al. 2022a).

### Phenotypic similarity

For each variant in a given gene, we calculated the Phenotypic Similarity between that gene and the proband’s phenotype. Computational analysis of phenotypes can greatly enhance the prioritization of variants. By computing measures of similarity between a patient’s clinical manifestations (provided as a set of HPO terms) and disease descriptions associated with genes, phenotype-based prioritization tools utilize standards and quantitative similarity measures to cluster and compare phenotype sets.

Phenotypic similarity scores are based on Human Phenotype Ontology information. Starting from standard phenotypic similarity indexes (e.g.: HPOSim package (Deng et al. 2015)) and exploiting the terms distance in the Direct Acyclic Graph (DAG) of the ontology, we developed a set of phenotypic similarity scores that take into account both the frequency of HPO terms in diseases and the specificity of the mutated genes in explaining sample’s phenotypes. More in detail, we firstly defined the HPO term sets associated with the gene and with the condition related to the gene as reported in Human Phenotype Ontology resources. We then exploited Resnik similarity between each HPO term of the patient and each term associated with the gene (or condition). Finally, we computed the phenotypic similarity between the two HPO sets, using the best-match average strategy (BMA), which calculates the average of all maximum similarities on each row and column of the similarity matrix S, as described in Eq. 1.

1 $$Sim_{BMA} \left( {g1,g2} \right) = \frac{{\mathop \sum \nolimits_{i = 1}^{m} max_{1 \le j \le n} s_{ij} + \mathop \sum \nolimits_{j = 1}^{n} max_{1 \le i \le m} s_{ij} }}{m + n}$$

Equation 1: best-match average strategy to compute the similarity between 2 sets of HPO terms. *g*1 of size m includes HPO terms provided to describe the patient; *g*2 of size n includes HPO terms associated with the gene (or with the condition). *s*_ij_ is the Resnik similarity between i-th HPO term of *g*1 and j-th HPO term of *g*2.

The frequency of each HPO term in the condition (if reported in HPO resource) is used to weight each HPO term contribution in *g*2, when combining Resnik similarities in Eq. 1.

The final phenotypic score is a combination of the gene-based and condition-based phenotypic similarities computed as described above.

We performed a preliminary analysis on 35 training set samples to evaluate the phenotype-based prioritization capabilities of our phenotypic score against other common solutions. We evaluated the prioritization performance of various tools (Phen2Gene (Zhao et al. 2020), Amelie (Birgmeier et al. 2020), Phrank (Jagadeesh et al. 2019) and Phenolyzer (Yang et al. 2015)) by examining the ranking of the causative gene based on its phenotypic similarity with the clinical phenotypes presented by the patient. A list of all mutated genes and HPO terms was given as input to each of the tools. The computation of the phenotypic similarity score is limited to genes with known associations with phenotypes, according to the HPO database.

### Inheritance information

To encode inheritance information for each variant, we consider three key factors: the genotype of the variant, the expected inheritance mode of the disorder (e.g.: as reported in MedGen for the gene where the variant is located), and any available family segregation data (Licata 2023). Briefly, the expected mode of inheritance for each variant (such as autosomal dominant or recessive, X-linked, or de novo), is assigned based on the family segregation pattern (if available) and the variant’s genotype (Table 2). Secondly, we evaluate the expected inheritance mode of the condition associated with the gene where the variant is located. This evaluation is based on the inheritance mode reported in MedGen for the gene. If these two values match, this information is used to trigger a binary feature indicating whether the variant is inherited or not.

**Table 2:** Inheritance pattern description and assignment’s rules when family analysis is available

Inheritance pattern description	Triggering rule
Autosomal/ X-linked/ Y-linked de novo variants	Heterozygous variants in the proband(s), but not present in the healthy parents
Autosomal/X-linked recessive compound heterozygous variants	At least two heterozygous variants in the proband(s) in the same gene, inherited from healthy parents (or de novo)
Autosomal/X-linked homozygous recessive variants	Homozygous (or Hemizygous in case of chrX for males) variants in the proband(s), inherited from healthy parents
Autosomal/X-linked isodisomy	Homozygous variants in the proband(s), inherited from only one healthy parent, having the same variant in heterozygous
Y linked	ChrY variant in the male proband(s) inherited from the affected father

### Variant quality

Lastly, the quality of each variant is assessed and encoded. Higher-quality variants (as an example, in terms of filter, genotype quality, allelic balance, and coverage), are generally more reliable and carry stronger implications for disease association and inheritance.

**Fig. 1:**
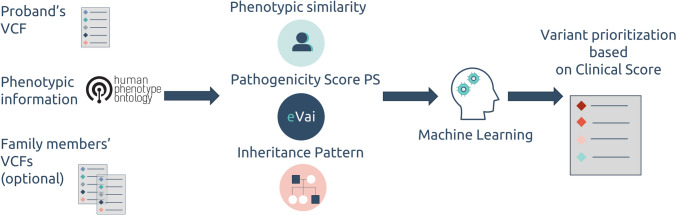
Workflow of the “Suggested Diagnosis” model

According to this description, in the dataset built for the CAGI challenge, each instance represents a possible genetic diagnosis (a single variant or a combination of candidate compound heterozygous variants) and the features cover the four described levels of information. This feature had a dichotomous value (0/1), computed based on the value of the filter field reported for each variant. Specifically, a filter field value of “Pass” was indicated as Quality = 1, while any value other than “Pass” was indicated as Quality = 0 (Fig. 1).

### Training and evaluation strategy

The labeled dataset provided by the organizers consists of 35 solved cases (*training set*). Each training sample contains a median of 7700 variants, which were included in the training set, totaling approximately 269,000 variants overall. Among all these instances, the causative variants indicated by the data providers were labeled as pathogenic, while the remaining variants were labeled as benign. A detailed description of the 35 cases is reported in the CGI6 RGP Challenge publication (Stenton et al. 2023).

The decision to solely utilize the training set provided by the CAGI organizers as a source of information during the training phase is driven by the aim to maintain homogeneity between the training and test sets. These two provided datasets exhibit homogeneity in terms of experimental factors such as sequencing platforms and genomic targets. Additionally, they show similar overall variant numbers and distributions, including coding/non-coding variants proportion. The phenotypic descriptions are consistent and performed by the same pool of clinicians, eliminating any phenotype selection bias between the training and test sets. Furthermore, the distribution of features, such as pathogenicity scores and quality measures, is similar, as shown by the reliability analysis that we performed (described in the following section). However, to exclude the possibility that the model overfits the CAGI data, we assess the generalization capabilities of the model on an additional independent test set (the DDD dataset), described in the next paragraphs.

For the solved test cases, the true causative was not made available, to compare the performance of different teams in an unbiased way. Therefore, to perform model selection and to preliminary understand whether our approaches work well, we set up a Leave-One-Proband-Out Cross Validation (LOPO CV) on the training set. In detail, for each of the 35 training probands, we retained that proband for testing (LOPO test proband, containing a median of approximately 7700 variants), and we trained different models on the remaining 34 probands (training set with a median of approximately 261,800 variants). We evaluated the ranking performance on the LOPO test proband that was not included in the training. This procedure was repeated for each training probands, thus resulting in 35 trained models for each different machine learning classifier and 35 evaluated cases. Given the phenotypic heterogeneity characterizing the 35 probands, the LOPO strategy allows the assessment of the generalization capabilities of the machine learning models.

We compared the performance of different machine learning classifiers, all implemented in the scikit-learn package (Pedregosa et al. 2011), such as Bayesian classifier, linear models, ensemble models, and Multilayer perceptron. The best models were then trained on the complete training set and were used to predict the 30 test cases provided by the CAGI organizers.

### Predictive uncertainty and reliability analysis

In addition to predicting the probability that each variant is disease-causing, CAGI organizers admit that also a standard deviation of the prediction can be submitted. Some classifiers, such as Bayesian classifiers, already embed a notion of uncertainty, while to calculate the uncertainty of the prediction for the ensemble models we exploited the framework suggested by Shaker et al. (Shaker and Hüllermeier 2020): we computed the uncertainty as Shannon entropy of the “weak” classifiers’ predicted probabilities, where the “weak” classifiers are the classifiers in the ensemble.

We’ve addressed this task by computing the uncertainty of predictions through the analysis of its reliability.

Machine learning generalization ability represents the ability of the classifier to maintain good performance not only on train/development data but also on different datasets that can be provided during deployment over time. Poor generalization ability of machine learning models in time and across different datasets has been widely reported, especially in healthcare, and it can hamper trust in machine learning prediction (Kelly et al. 2019). A possible cause for a decrease in performance is *dataset shift* when the variable distributions greatly differ from training. In the context of variant interpretation, it has been shown that the performance of in silico tools to predict variants’ damaging impact greatly varies and is affected by *circularity* and *error propagation*, when the same genes exploited to train the models are also used to evaluate them during tests (Grimm et al. 2015). Consequently, machine learning performance can decrease on new data. To understand if the prediction of a model trained on a particular set of data can be considered reliable, i.e. if we can trust such prediction, a reliability assessment can be performed. With the term “pointwise reliability” we refer to the degree of trust that a single prediction is correct. A simple criterion to determine whether a prediction may be reliable is the so-called “density principle”, that checks if the classified instance is similar to the training set. If so, we are more willing to trust the prediction in this instance, because the model may have successfully learned to classify data in the same features space and distribution of the training set (Nicora and Bellazzi 2020). This approach remains completely independent of the ground truth class of the tested instance, which is unknown in a real deployment case, as well as in the context of the CAGI 6 RGP challenge. Instead, it relies only on feature distributions. To ensure the trustworthiness of our models’ predictions, we implemented a reliability assessment framework capable of establishing if the test cases originate from a population similar to the training cases, and therefore if we could trust the predictions of our models. Specifically, we compare the feature distributions of the training set with those of the test set, employing a previously developed reliability assessment approach (Nicora and Bellazzi 2020; Nicora et al. 2022b). Briefly, for each feature, we calculated the “borders” in the training set (Olvera-López et al. 2010). Borders are defined as training instances in a class that exhibit the closest proximity to an example of the opposite class, for a given attribute, or that have the minimum/maximum value for that feature. For each variant in a test case, we compare its attributes with the corresponding training borders, and we record the number of attributes for which that variant would become a border if included in the training set. The higher this number, the more information this case would add to the training, and therefore the less it is similar to the current training. The reliability score is calculated as the following formula:

$$rel\left( x \right) = 1 - \frac{{m_{x} }}{m}$$

where ${m}_{x}$ is the number of attributes for which the instance would become a border and $m$ is the total number of attributes. Therefore, for each variant in each test case, we assign a reliability score from 0 to 1 according to the above formula. Scores closer to 1 indicate a higher degree of reliability. For each test proband, we calculated various statistics, such as the median and standard deviation of the reliability score, the minimum reliability score, and the percentage of variants that have a reliability score equal to 1.

## Results

### Phenotype-based gene prioritization

**Fig. 2:**
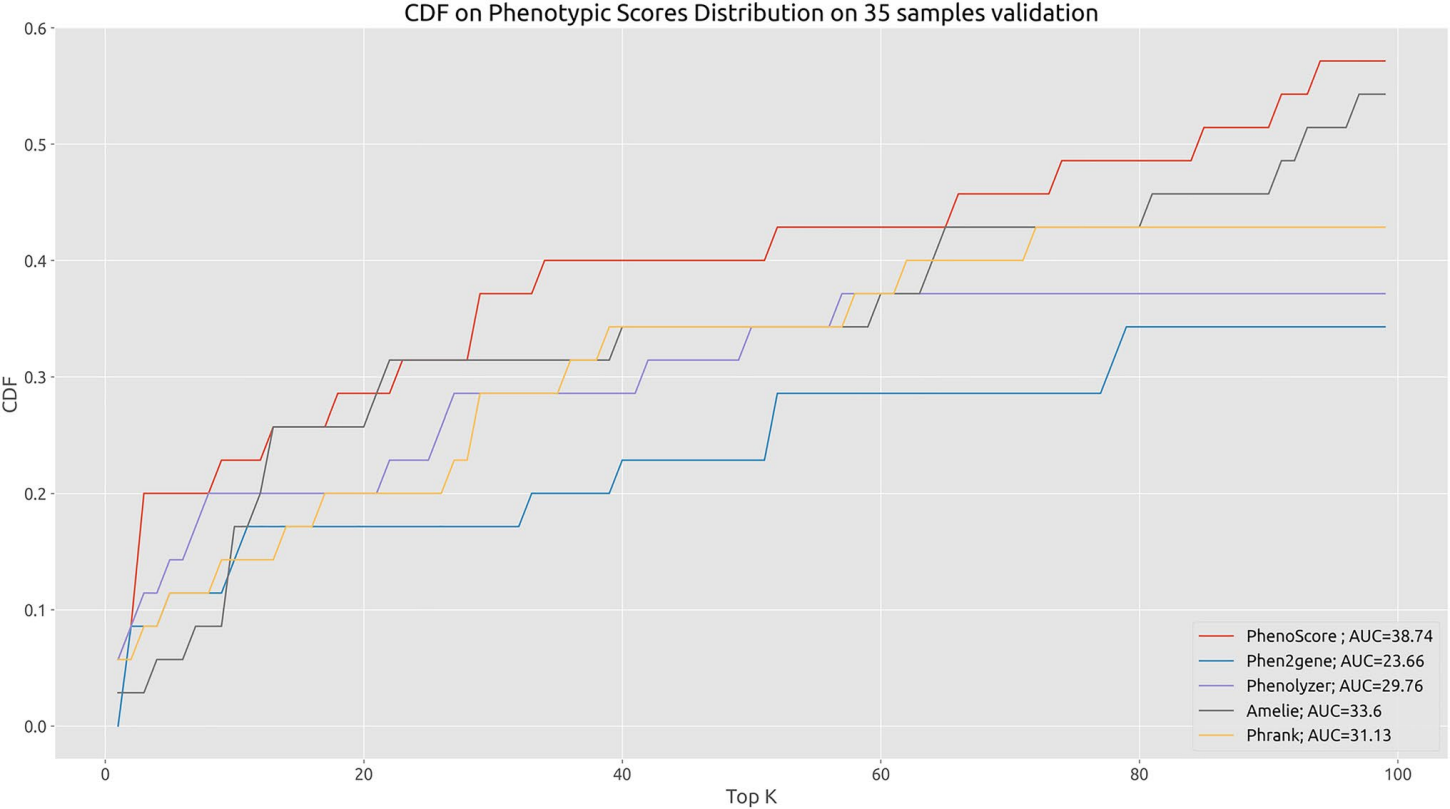
Cumulative distribution function (CDF) of phenotype-based prioritization performances on 35 training set samples. PhenoScore represents the implementation of the Phenotypic Similarity score described in the Methods section

The phenotypic score exploited in our model was compared with other published phenotypic similarity metrics. On the testes dataset, it showed a better performance in terms of ranking of the causative gene compared to Phrank, Phen2Gene, Phenolyzer and Amelie (Zhao et al. 2020; Birgmeier et al. 2020; Jagadeesh et al. 2019; Yang et al. 2015). However, as highlighted by the Cumulative Distribution Function in Fig. 2, even if our phenotypic similarity measure performs slightly better than the others, it cannot be solely exploited to consistently rank the causative gene in the topmost positions, since the causative gene is prioritized in the top 100 positions only in 58% of cases. At least the contribution of the variant impact needs to be taken into account to achieve decent prioritization performances. A comprehensive approach able to integrate variant pathogenicity, family inheritance, and variant quality metrics might highly contribute to enhancing the prioritization performances.

### Dataset preprocessing and features selection

**Fig. 3:**
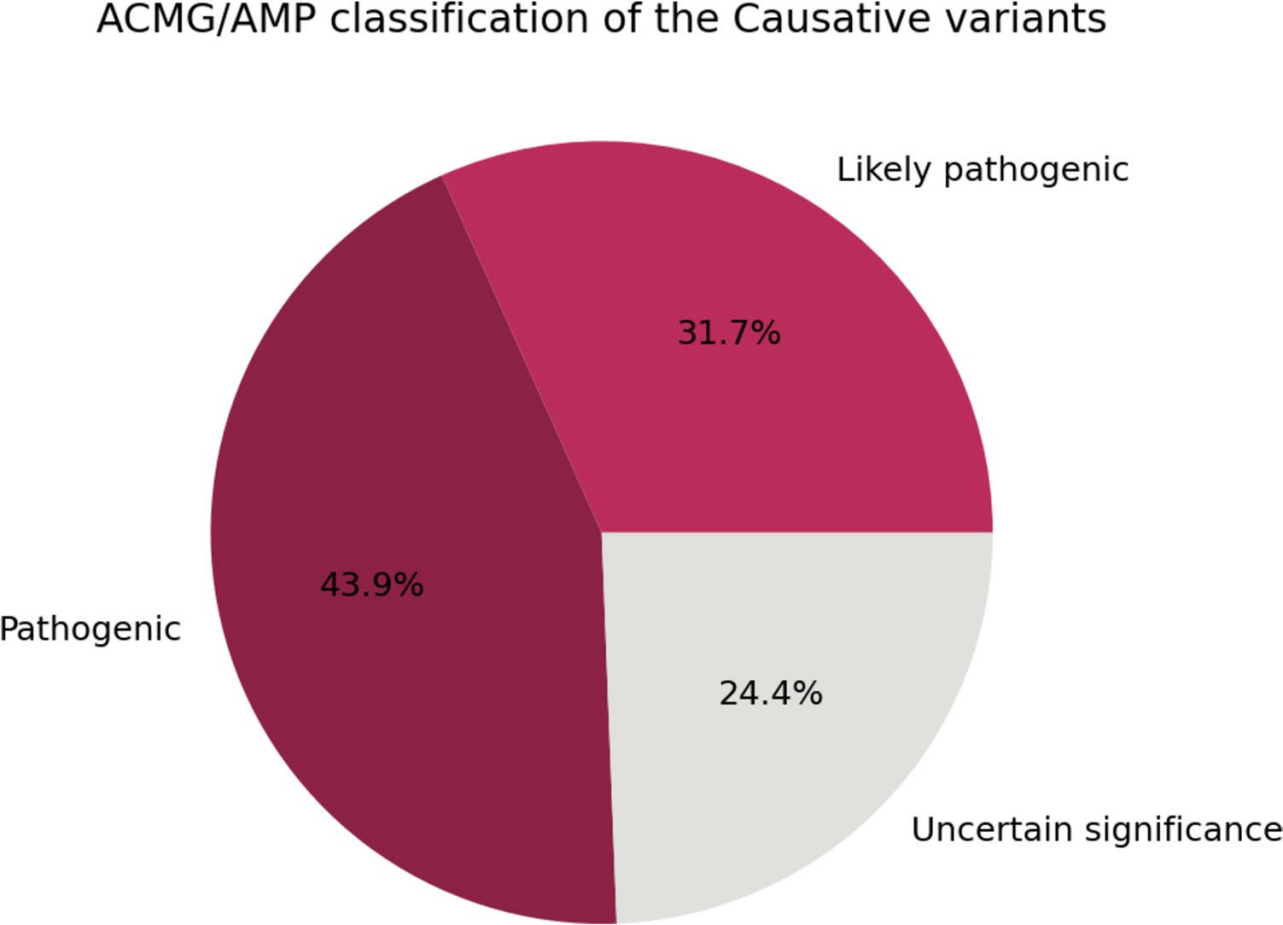
Percentage of causative variants in the training set (*n* = 35 solved patients) with their interpretation according to the ACMG/AMP guidelines as implemented in the eVai software (enGenome srl, Pavia)

We analyzed the causative variants provided in the training set in light of the ACMG/AMP guidelines (Fig. 3). Most causative variants (75%) are either Likely pathogenic or Pathogenic. The remaining are interpreted as VUS.

**Fig. 4:**
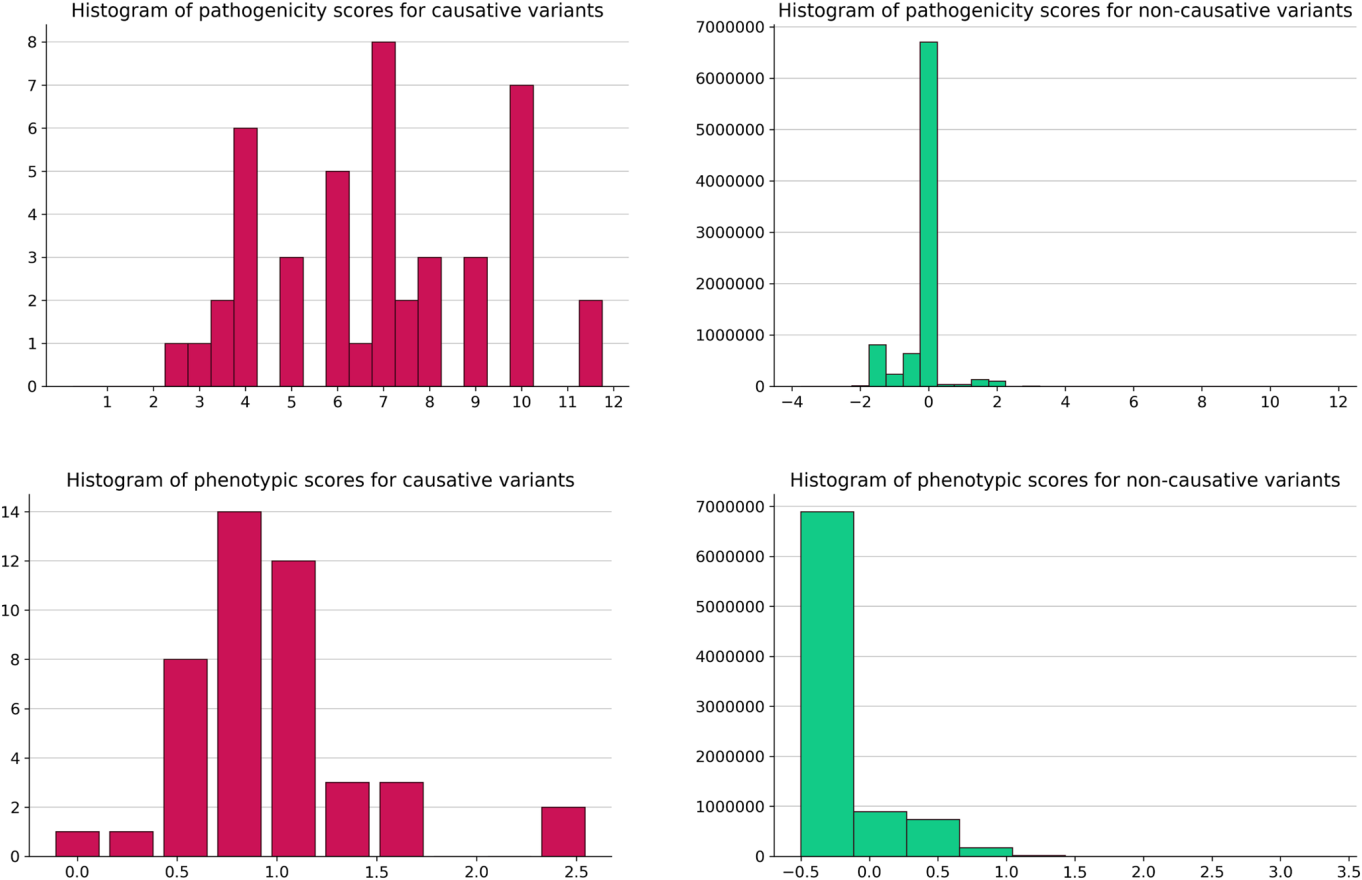
Distribution of pathogenicity score and phenotypic similarity score for causative and non-causative variants in training

Figure 4 shows the distribution of two features (pathogenicity score and phenotypic scores) on training variants. As we can see, causative variants have higher values both of pathogenicity and phenotypic scores, indicating that these two features can be highly informative to distinguish between causative and non-causative variants.

### Training and evaluation strategy

**Fig. 5:**
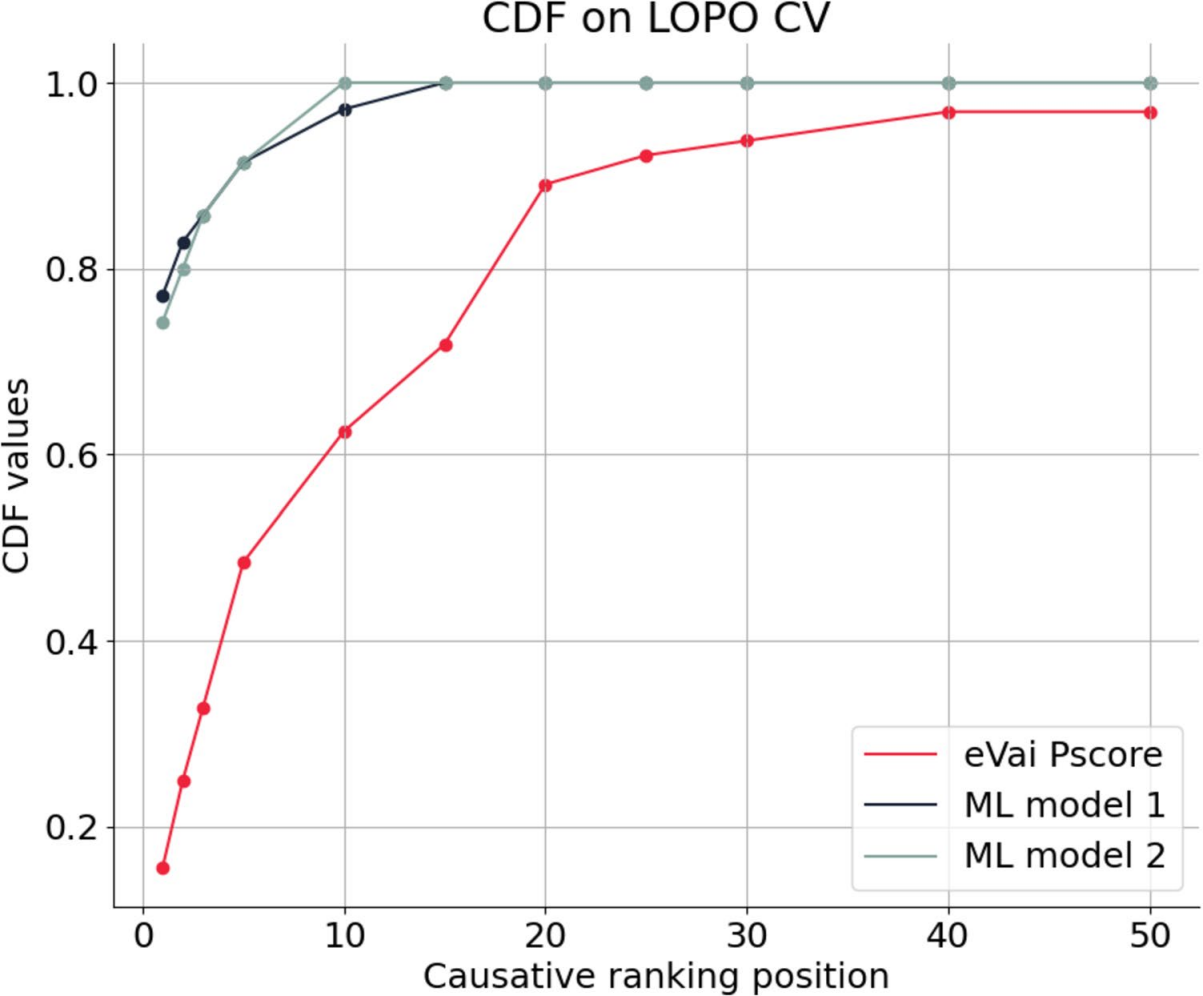
Cumulative Distribution Function (CDF) calculated with the LOPO CV on the training set

The best-performing models resulted to be an ensemble classifier (*ML model 1*) and a linear classifier (*ML model 2*). Figure 5 shows the Cumulative Distribution Function (CDF), which encodes the fraction of cases for which the causative variant is ranked at the top K positions, calculated on the 35 training cases in the LOPO CV. Not surprisingly, the eVai pathogenicity score, which is only based on the ACMG/AMP guidelines and is not a data-driven model, shows lower prioritization ability in comparison with the machine learning models that integrate both ACMG/AMP standard guidelines, phenotypic similarity, inheritance hypothesis, and variant quality information. In particular, the *ML model 1* ranks the causative in the very first position in 27/35 (77%) of cases, while the *ML model 2* ranks the causative in the first position in 26/35 cases (74%). 100% of the causative variants are ranked within the first 10 positions for *ML model 2*, while in one case the *ML model 1* ranks the causative variant in the 12th position.

### Predictive uncertainty and reliability analysis

**Fig. 6:**
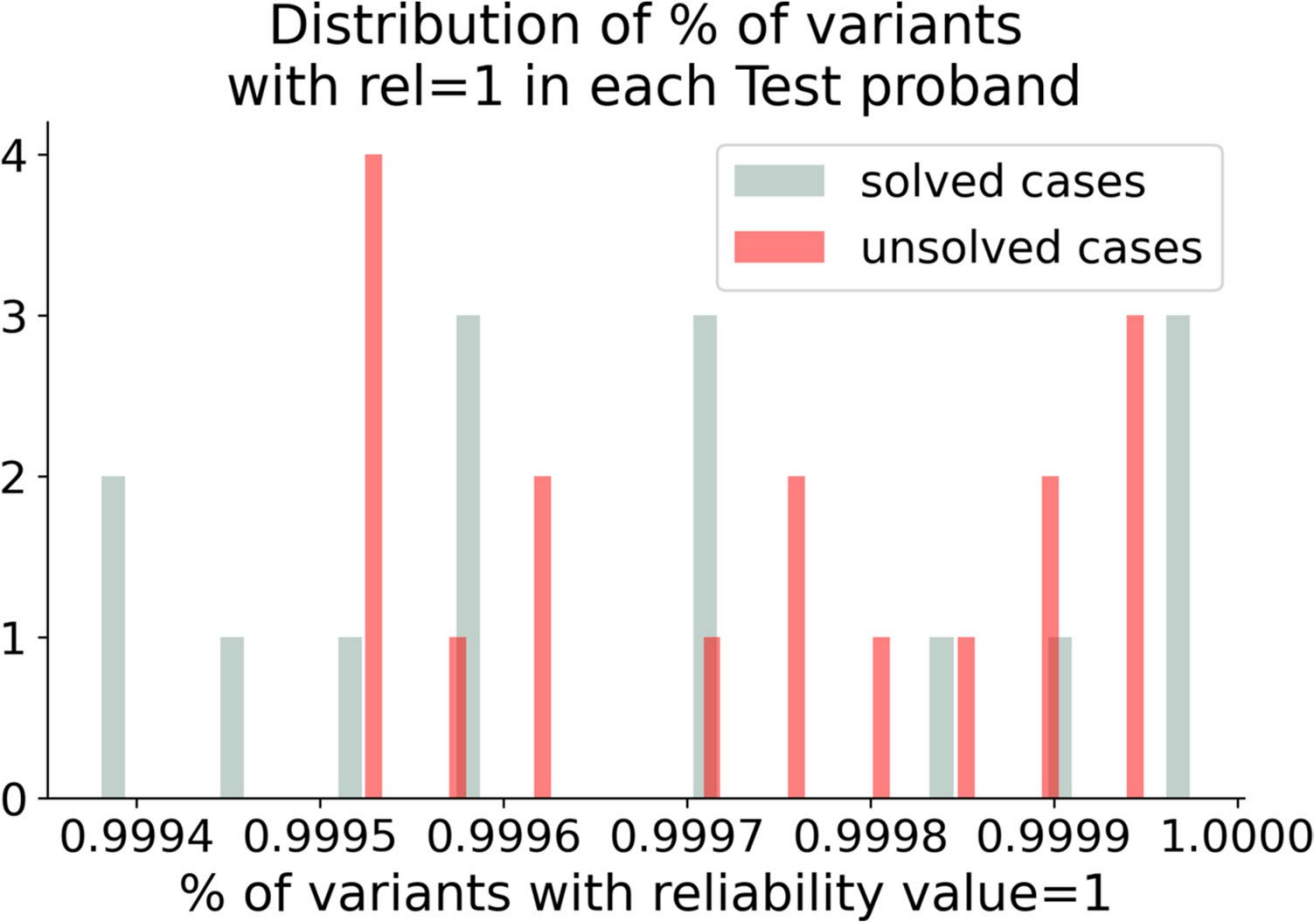
Histogram showing the percentage of variants in each test proband with reliability equal to 1. Both solved and unsolved test cases show high similarity with the training set in terms of our built-in features

Figure 6 indicates the distribution of the percentage of variants that have a reliability of 1 in each test case. In all the test cases the percentage of variants with maximum reliability is really high, above 99%. In five Test probands, all the variants have a reliability of 1. This result shows that our features set is consistent between train and test and that the train and test populations do not come from highly heterogeneous populations. Therefore, we could expect good performances from our machine learning models.

### Performance on the neurodevelopmental disorders

**Fig. 7:**
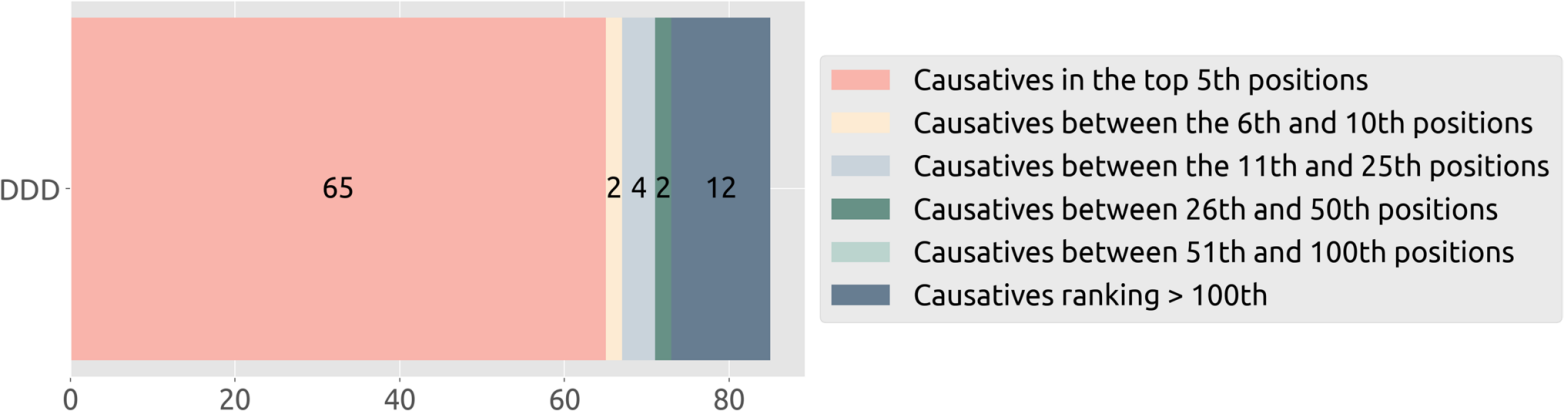
Number of causative variants in the DDD dataset ranked at different positions

We further assess the validity of our approach on an additional dataset, which is a subset of 85 samples of the “Deciphering Developmental Disorder” (DDD) study (Firth 2011; Deciphering Developmental Disorders Study 2015). We selected 35 challenging cases for which the causative variant has a low eVai pathogenicity score (0.88 mean pathogenicity score, with a minimum value of 0 and a maximum of 0.99, while the mean pathogenicity score in training causative is 0.99, spanning from 0.9955 to 0.9999) and 50 cases selected randomly. The Model 1, trained on the CAGI training cases, was used to rank DDD variants. In Fig. 7, the number of causative variants ranked at different positions is reported. For most cases (65/85 = 76%), the causative variant was ranked in the top 5th positions.

**Fig. 8:**
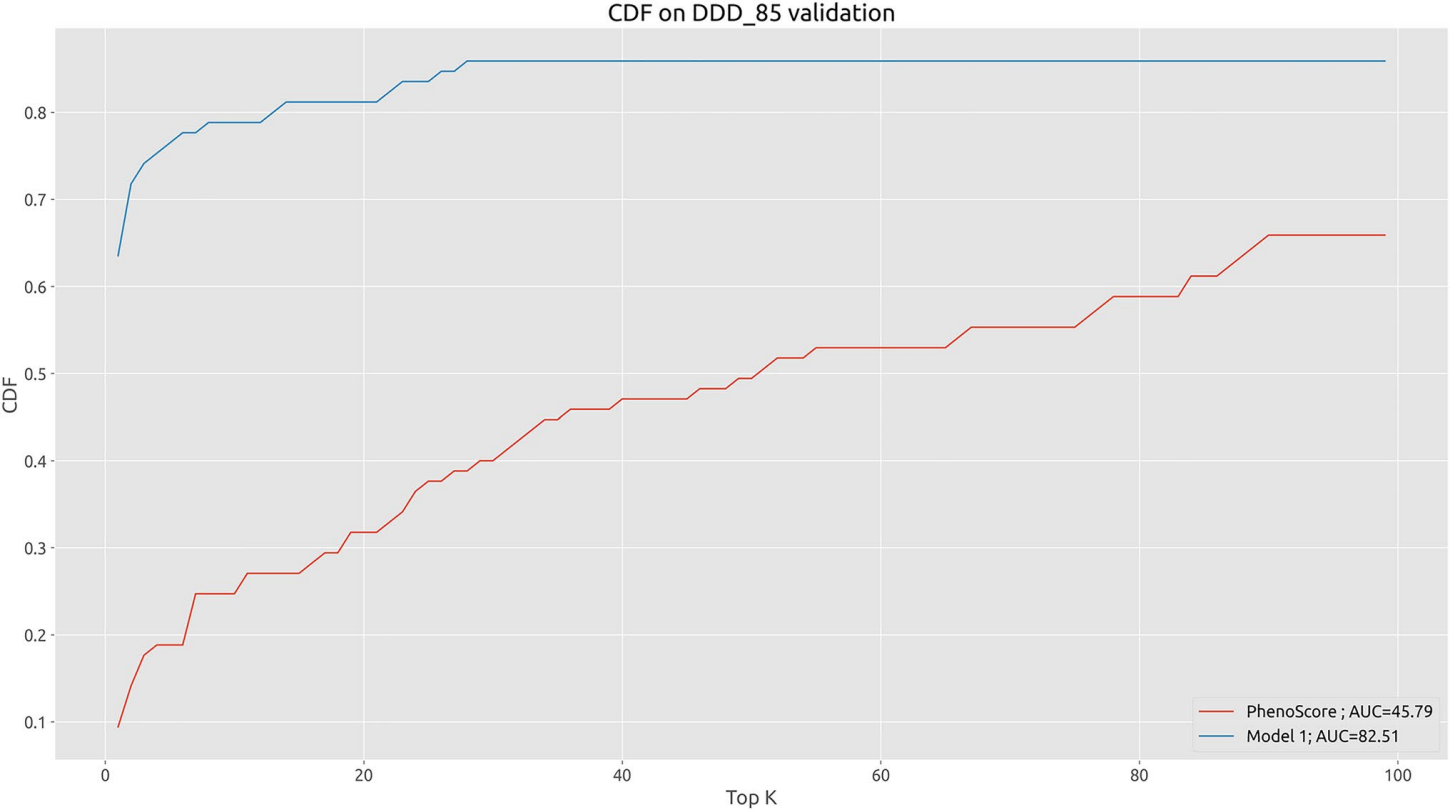
Cumulative Distribution Function (CDF) calculated on 85 DDD samples comparing Model to, eVai pathogenicity score and to phenotype-based only prioritization

Furthermore, we compared the prioritization capabilities of our model with the ones based on the phenotypic score only (Fig. 8). Exploiting the phenotype-based prioritization, the causative gene was ranked in the top 5th positions in 19% of the cases (16/85) compared to the 76% of the Model 1 prioritization.

## Discussion

Suggesting the causative variant for a Rare Disease patient is a compelling task and many data-driven approaches have been developed to fulfill it (Li and Wang 2017; Scott et al. 2019; Nicora et al. 2018; Whiffin et al. 2018; Xavier et al. 2019; Ravichandran et al. 2019; Peng et al. 2021; Kopanos et al. 2019). We have developed machine learning models that leverage standard guidelines for variant interpretation, phenotypic similarity, family information, and variant quality, to rank all the variants in a VCF file according to their predicted probability of being causative. This approach, named “Suggested Diagnosis”, has been benchmarked in the public CAGI6 challenge on real data from the Rare Genome Project (Stenton et al. 2023). Sixteen different teams participated in this challenge with 52 different models. Five of these teams were disclosed in a recent publication as participants in this challenge and their performance was publicly divulgated: Invitae Moon, Katsonis et al. (Katsonis and Lichtarge 2014), enGenome team, TCS group (Rao et al. 2020), and the Exomiser group (Smedley et al. 2015).

The enGenome team resulted in being a best performing predictor within the CAGI6 RGP challenge. Unlike other benchmarked tools, we included both coding and non-coding variants, even deep intronic ones, utilizing the entire dataset from Whole Genome Sequencing. In contrast, other groups limited their analysis to coding or near-splice variants. Among all the diagnosed patients analyzed in the challenge, the Suggested Diagnosis identified the causative variant as the top prioritized choice in 50% of cases and within the top 5 positions in 71% of cases. This demonstrates that prioritization performance remained consistent despite the increased number of variants in the analysis.

Regarding the four “difficult-to-predict” causative variants described by Stenton et al. the Suggested Diagnosis could not prioritize two of them as, at the time of the challenge, they had no known disease associations according to OMIM, MedGen, Disease Ontology, and Orphanet. However, the remaining two difficult-to-predict variants, which were associated with disease-associated genes, were correctly predicted and prioritized by the Suggested Diagnosis.

On the cohort of undiagnosed patients, the Suggested Diagnosis was the only predictor of the challenge able to identify the causative variants in two unsolved test cases, that were later validated as the true causatives and returned to the patients. In more detail, one of these cases involved compound heterozygosity with a frameshift variant and a deep intronic variant on the ASNS gene. Our model was the only one capable of identifying the deep intronic variant, while a few other models were able to predict only the frameshift variant as the first hit. In the second undiagnosed case, a de novo splicing variant on the TCF4 gene was identified by a total of 8 models, 4 of them based on our Suggested Diagnosis.

The “Suggested Diagnosis” model implemented by enGenome mimics the reasoning process of a geneticist during the variant evaluation process: variants’ pathogenicity, phenotypic overlap, inheritance fit and variant quality. Variant’s pathogenicity is based on the enGenome pathogenicity score (Nicora et al. 2018, 2022a).

The phenotypic overlap metric was compared with available solutions published in recent years 34–37. Although our score outperforms other tools in phenotype-based prioritization, this approach alone is insufficient to consistently rank the causative gene in the topmost position. During the development phase, we defined and carried out a “Leave-One-Proband-Out” cross-validation, that allowed us to use the training samples with known causative variants to unbiasedly select the best models for submission and to assess the generalization capabilities of the designed features for patients affected by heterogeneous disease and different phenotypic spectrum. Additionally, we performed a reliability assessment to preliminary understand whether we could trust the predictions on the CAGI test set. Subsequently, we further validated our approach to cases with neurodevelopmental disorders from the DDD study (Firth 2011). Furthermore, the comparison conducted on these 85 independent samples demonstrates that our hypothesis-driven approach, incorporating all four levels of information (variant pathogenicity, inheritance information, phenotype similarity, and quality of the variant), is substantially more effective than relying solely on phenotype-based prioritization. In summary, the training phase leverages 269,00 variants from 35 WGS proband data, with known causative variant. This approach was demonstrated to be reliable even with a training set containing a limited number of high-quality causative variants (35 in this case) and proved to have good generalization performance, thanks to the analysis of the DDD dataset. After the challenge was concluded, the “Suggested Diagnosis” has been retrained on in house data and is now available within the eVai software.
